# Three Intervention Programs in Secondary Education on Attitudes Toward Persons With a Disability

**DOI:** 10.3389/fpsyg.2022.787936

**Published:** 2022-05-19

**Authors:** Julián Álvarez-Delgado, Benito León-del-Barco, María-Isabel Polo-del-Río, Santiago Mendo-Lázaro, Victor M. Lopez-Ramos

**Affiliations:** Departamento de Psicología and Antropología, Facultad de Formación del Profesorado, University of Extremadura, Badajoz, Spain

**Keywords:** attitudes, adolescents, disability, intervention, secondary education

## Abstract

Persons with a disability make up a social group which is in an especially vulnerable situation. They have to face obstacles and difficulties in their participation as part of the community with equal opportunities, in which attitude of others is a determining factor. This study makes a comparative analysis of three intervention programs (1 “Simulation and Modeling,” 2 “Information and Awareness Raising,” and 3 “Adapted Sport”) on attitudes toward persons with a disability of adolescents in secondary schools. Each program is based on a concrete technique, but they all have the common thread of the direct, structured contact technique with persons with a disability. The effectiveness of the three programs in changing attitudes is analyzed, and their impact on the different factors of the attitude construct (1 “acceptance/rejection,” 2 “competence/limitation,” and 3 “equality of opportunities”) is also studied. The results show the effectiveness of the three programs. The students show more positive attitudes toward persons with a disability in all the groups, especially program 1. Analyzing the general influence of the three programs on the factors of the attitude construct, it can be seen that in factors 2 and 3, the attitudes have significantly improved in all three programs. Finally, the results show that each program has been more effective on a concrete attitude factor.

## Introduction

Certain groups of people in our society find themselves in a situation of special vulnerability, defined by the identification of certain personal characteristics that define them as a group and which differentiate them from the current stereotype of normality. These groups have to face obstacles and difficulties to their participation in the community as they do not enjoy equal opportunities with respect to the rest of the people, in contradiction to their rights as should be guaranteed by an adequate system of recognition and protection of human rights (Campoy, 2017). The Convention on the Rights of Persons with Disabilities (Organización de las Naciones Unidas, 2006), as the principal instrument of the United Nations to protect the rights and the dignity of persons with disabilities, sets out as the fundamental principles respect for a person’s inherent dignity, an absence of discrimination, the full and effective participation and inclusion in society, the respect for difference and the acceptance of the collective as part of the diversity, and equal opportunities, among others.

According to the Organización Mundial de la Salud (2011), a disability is understood as the interaction of different factors and deficiencies, including limitations to an individual’s activity as well as restrictions to his/her participation. Before reaching this current vision of disability, a long period of adaptation has passed during which our understanding focused on the deficiencies and limitations of the person and his/her lack of abilities (medical-rehabilitation model). In the 1970s, a new way of understanding disability started in the United States, creating the foundations for the future movement for an independent life (DeJong, 1979; Shapiro, 1994). At the same time, in the United Kingdom, there arose, from a sociological viewpoint, the development of the social barriers model, which would later take the form of the social disability model (Barnes et al., 2002). Both movements have a new vision of disability, one which stresses the importance of the social barriers as a decisive factor in the limitations and difficulties that this collective suffer. From such a viewpoint, a disability ceases to be defined as a person’s physical, mental, intellectual, or sensorial limitation, becoming instead a social limitation. The social model sees a disability not only as an attribute of a person, but also as the product of the interaction between a set of conditions, many of which are created by the social environment (Hasler, 2003; Ferreira, 2008; Lawson and Beckett, 2021). Such a focus opens the way for a new concept centered on human rights and posing a change of paradigm in the way of understanding a person with a disability.

A person’s disability and her/his functioning are conceived as a dynamic interaction between the state of health and contextual factors, which also involves both personal and environmental factors which make a full and effective social participation in conditions of equality with other persons more difficult (Organización Mundial de la Salud, 2001; Organización de las Naciones Unidas, 2006; Abellán and Hidalgo, 2011). Among these said factors, it is worth mentioning the attitudes toward the collective because of their great influence. Such attitudes are a determining relational and environmental factor which can either facilitate of hinder the process of inclusion in society (García and Hernández, 2011; Organización Mundial de la Salud, 2011). For Luque and Luque-Rojas (2011), discrimination toward persons with a disability may derive in social exclusion, which can finally lead to a loss of their rights and active participation in society.

Thus, as the relation between a person with a disability and his/her environment is vital, then the attitudes (understood as the social constructs of those who surround them) play a fundamental role in the development and future of the person and her/his process of inclusion (Hampton and Xiao, 2008; Bešić et al., 2017).

The construct attitude understood as an idea charged with emotion that predisposes one toward a particular kind of actions when faced with a certain stimulus (Triandis, 1971), refers to the three-dimensional model of attitude built up around three components: the cognitive, which is made up of beliefs (stereotypes); the affective, which is made up of the emotions (prejudice); and the behavioral, following the tendency or predisposition to have negative, discriminatory behavior patterns toward members of the minority group that tend to marginalize them (discrimination; Rosenberg and Hovland, 1960; González and Leal, 2009; León et al., 2009). Upon this basis, society still manifests itself through disaffected social beliefs and associated behavior patterns, predominantly negative and pejorative attitudes that stigmatize and exclude persons with a disability, undermining their full inclusion in society (UNICEF, 2013; Sevilla et al., 2014; Echeverría and Galaz, 2018). Depending on whether the attitudes are positive or negative, they either favor or reduce the capacity for inclusion on a social, family, or individual level (Rillotta and Nettelbeck, 2007; Shannon et al., 2009).

These attitudes form a determining contextual, environmental, and relational factor that makes the lives of certain persons or collectives more difficult in all areas. Thus, one of the main barriers that persons with a disability must face is not the disability in itself, but the lack of acceptance on the part of others, caused by ignorance, indifference, and unease (Behler, 1993; Miller and Cordova, 2002). Among the negative attitudes toward disability, the term “ableism” has arisen to define a form of discrimination or social prejudice, oppression, and abusive behavior that arises from the belief that persons with a disability are inferior to others (Nario-Redmond, 2010; Nario-Redmond et al., 2019).

One of the expressions of “ableism” is micro-aggressions, a very common social phenomenon concerning persons with a disability (Moral et al., 2020). These micro-aggressions affect all spheres of life, occurring in such areas as the family, school, and even the disability organizations themselves (Oliver, 1990), and are perpetrated in a more or less disguised manner (Campbell, 2008, 2009; Wolbring, 2008; Bell, 2015; Bogart and Dunn, 2019). Thus, as mechanisms of discrimination, micro-aggressions interrupt the enjoyment of equal rights or make it impossible, creating a negative impact in the development of the self-concept and identity of the persons who suffer the said discrimination (Keller and Galgay, 2010; Dunn and Andrews, 2015; Forber-Pratt et al., 2017; Dirth and Branscombe, 2018, 2019; Harder et al., 2019). These stereotypes and prejudices that become discriminatory behavior (micro-aggressions) toward the collective are indications of the need to carry out awareness raising programs to bring about an effective and realistic change in attitudes.

Due to all of the above-mentioned points, attitudes are considered to be one of the most powerful barriers against the acceptance of persons with a disability, as well as being one of the biggest obstacles that exist in their path toward inclusion (Hampton and Xiao, 2008; Novo-Corti et al., 2011; Organización Mundial de la Salud, 2011). A growing number of intervention programs have recently been developed to modify and reverse the said prejudices and stereotypes that still exist concerning persons with a disability (Polo et al., 2011; Serrano et al., 2013).

The educational sphere is a privileged stage from which to enable a change in attitudes and to encourage positive attitudes toward persons with a disability (Shannon et al., 2009; Gurdián et al., 2020).

According to several works of research, attitudes are subject to constant change. Attitudes are learnt, in particular, during the first years of life when one’s attitudes are not so deeply rooted and it is here that education, through awareness raising programs, can favor positive changes in attitudes toward disability (Triandis, 1971; Guitart, 2002; Aguado et al., 2008; Cameron et al., 2011; Linsay and Edwards, 2012). Adolescence is considered to be a crucial stage in human development, when behavior patterns and attitudes are malleable and in a state of constant formation (Hurlock, 1971). When evaluating the attitudes of adolescent students toward their peers with disabilities, several works of research have confirmed that negative attitudes are held at these ages, being clearly defined at an earlier age (Aguado et al., 2004). They can even progressively worsen at the start of adolescence, paving the way in adulthood for such attitudes to become ingrained and irreversible (Blos, 1979; Muratori et al., 2010). So, it is relevant for this type of intervention to be encouraged at an early age in order to avoid preconceived attitudes toward persons with a disability (Víquez et al., 2020).

The intervention programs created to foment a change in attitude toward persons with a disability, developed both in Spain and outside Spain, all provide results which suggest that attitudes toward this collective are not as positive as one could hope and that they can be improved through conveniently planned intervention programs (Pérez-Tejero et al., 2012; Alcedo et al., 2013; Santana and Garoz, 2013; McKay et al., 2015; Harrison et al., 2019). Faced with this reality, the study of and research into the strategies and techniques included in the most effective attitude-changing intervention programs take on greater importance (Delgado, 2015).

Outstanding among the different theoretical approaches adopted to understand and explain the acquisition and modification of attitudes are the theories of consistency and contact. The first is based on the principle that persons make an effort to maintain some kind of coherence in their beliefs, attitudes, and behavior (Briñol et al., 2003). The second establishes that intergroup contact implies clearly defined, real, face-to-face contact between the members of the groups and tends to produce changes in attitudes (Allport, 1979; Pettigrew and Tropp, 2006; Lemmer and Wagner, 2015; Boin et al., 2021; Polo et al., 2021; Sadziak et al., 2021); giving rise to what Pettigrew (2009) called the “transfer of the secondary effects of contact,” in reference to the fact that those attitudes acquired through experiencing contact with a particular group can be transferred toward other groups not involved in the original situation.

As for the theoretical approaches that focus on contact and which are based on scientific evidence, some studies that investigate prejudice as the basis of discriminatory attitudes toward persons with a disability classify the contact theory as being among the principal focuses for reducing such attitudes (Paluck et al., 2019; Boin et al., 2021). This theory has given rise to a series of techniques for modifying attitudes toward persons with a disability, in particular the contact technique. In this sense, such systematic reviews as those of Pettigrew and Tropp (2006), Florez et al. (2009), and Lemmer and Wagner (2015) analyze the conditions that increase the effectiveness of the contact technique in changing attitudes. They stress: careful planning of the interactions and the encouragement of an intergroup climate that makes the individual skills of the persons with a disability more visible; gratifying interactions in an atmosphere of respect based on cooperation; the selection of persons with a disability based on criteria concerning motivation for one’s actions, credibility, competence in the areas of knowledge to be worked on, domination of social skills, and a positive attitude toward their own disability, while being willing to talk about it; prior training in the activity to be carried out so they can feel confident and safe, carrying out interactions based on the person’s skills and the disabling environment, instead of on the deficiencies and medical diagnoses of the disability, as well as on the preparation of the students prior to the interaction by indirect means (videos, readings, etc.).

The effectiveness of the contact theory is due to the fact that it facilitates the discovery of the positive attributes of persons with a disability through interventions based on structured contact and positive interactions which allow one to recognize the potential of a person with a disability, thus generating more accurate expectations and helping to eliminate barriers and prejudices toward them (Verdugo and Arias, 1991; Alcedo et al., 2013; Felipe et al., 2020). Intergroup contact has a great potential for effective interventions aimed at reducing prejudices (Boin et al., 2021).

Several works of research confirm the fact that prior, direct contact with persons with a disability can bring about an improvement in the attitudes toward this collective, as long as the said contact implies acquiring greater knowledge of the disability (Suriá, 2011; Armstrong et al., 2017; Abellán, 2018; Scanlon et al., 2020). Research into this question has shown that, when students receive information concerning persons with a disability and they also participate in significant interaction with them, more positive attitudes are seen (Rillotta and Nettelbeck, 2007; McKay et al., 2015). However, the success rate of the reduction in prejudicial attitudes is not limited to direct interventions; later studies have shown that successful interventions can also be carried out through diverse forms of indirect contact, such as vicarious, imagined, or virtual contact (Lemmer and Wagner, 2015; Cocco et al., 2020).

In the educational context, certain documentary reviews and analyses of attitude changing programs analyze the procedures used in their implementation. Here, the information is presented in the form of discussion groups and work groups, with simulations and role playing the condition of the disability in question. There is also direct contact with persons with a disability, indirect contact through videos and films, imagined contact, through activities involving reflection and practical experiences, as well as teaching ways to conceptualize disability. Such are the strategies that can be seen to have the greatest effectiveness in attitude reorientation programs (Lean et al., 2006; Linsay and Edwards, 2012; Santana and Garoz, 2013; Martos-García et al., 2016; Árnadóttir et al., 2018; Logan and Bogart, 2020; Reina et al., 2020; Kirk et al., 2021).

The technique of simulation and modeling involves the vicarious experience of observing the simulation of a disability, in order to positively modify attitudes (Stathi et al., 2014; Ginevra et al., 2021). Several studies consider it to be one of the most effective strategies for promoting changes in attitudes (Slininger et al., 2000; Hodge et al., 2002; McCarthy and Light, 2005). In this sense, a greater benefit is noticed if the subjects who experience the simulation perceive the reactions of the persons with a disability and the reactions toward themselves as simulators (Donaldson, 1987; Horne, 1988). According to certain studies, the technique is most effective if the experience is planned with sufficient time to provide the participants with the understanding of the effort required by those with a disability to overcome the challenges that the disability on occasions presents, taking on the activity seriously so it raises their awareness of what persons with a disability could do if they had the appropriate adaptations available. Furthermore, if after completing the activity, there is a discussion/debate concerning the experience they have had (French, 1992; Kiger, 1992; Verdugo et al., 1994; Herbert, 2000; Burgstahler and Doe, 2004; Hurst et al., 2012).

However, there are studies that question this technique, either because the empirical evidence sustaining it as a technique to foster changes in attitudes is weak, or because certain risks are associated to it for the participants, such as stress, pressure or a lack of confidentiality (French, 1992; Kiger, 1992; Herbert, 2000; Nario-Redmond et al., 2017). Along these lines, other studies indicate that the simulation of disabilities generates anxiety while not promoting any improvement in attitudes toward persons with a disability, thus affecting the collective’s integration into society. Other studies raise the question that simulation as a technique supposes living through the difficulties of other persons and that, in the sphere of physical disability; it can be counterproductive, as it diminishes the perceived competences of the persons with a disability (Silverman et al., 2015).

As for the technique of awareness raising and information, it should be said that the progress made in overcoming psychosocial barriers have been achieved through intervention programs whose most commonly used techniques are awareness raising and information concerning the disability (Gurdián et al., 2020; Lawson and Beckett, 2021). Research in this respect has shown that when students receive information concerning persons with a disability and also participate in significant interactions with them, they then reflect more positive attitudes (Rillotta and Nettelbeck, 2007; McKay et al., 2015). Research into this question has shown that, when students receive information concerning persons with a disability and they also participate in significant interaction with them, more positive attitudes are seen (Rillotta and Nettelbeck, 2007; McKay et al., 2015). Greater knowledge of a disability can lead to improved attitudes in the long term (Navas et al., 2004; Vignes et al., 2009; Moore and Nettelbeck, 2013); while ignorance and a lack of information can negatively affect students’ attitudes (Nowicki, 2006; Rillotta and Nettelbeck, 2007; Ison et al., 2010; Linsay and Edwards, 2012). Recent studies have stated that, in order to develop interventions which can generate positive attitudes, it is necessary to design awareness raising programs that consider direct contact with persons with a disability (Ocete et al., 2022). Prior contact helps to reduce intergroup anxiety which, together with increased empathy, can act as mediators for the effects of contact (Pettigrew and Tropp, 2006; Seger et al., 2017).

As for the technique of adapted sport, a change in attitudes toward persons with a disability has been demonstrated in those who participate if it is combined with information concerning the disability and the adapted sports, a simulation of the disability in the said sports, debates and guided discussions (Guitart, 2002; Aguado et al., 2004; Santana and Garoz, 2013). This technique also supposes a contribution to education in values and the social awareness raising of those who participate in the said technique (Robles-Rodríguez et al., 2017). According to Abellán et al. (2022), one of the commonest forms of simulating a disability in Physical Education is through the practice of adapted sport. Although there is a scarcity of scientific publications concerning intervention programs with persons with a disability in the sphere of physical sporting activity or studies concerning attitude changing programs in school populations that use physical sporting activity as a fundamental guide (Felipe and Garoz 2014; Felipe, 2017); positive results are considered in some of them with respect to changes in attitude in school populations and young people (Xafopoulos et al., 2009; Reina et al., 2011; Pérez-Torralba et al., 2019).

Thus, this study aims to analyze which intervention modes are most effective in combination with the contact technique for generating changes in attitude toward persons with a disability in the adolescent population in schools. We also aim to analyze the effect of the said techniques on the different components of the attitude construct. To be more precise, we carry out three intervention programs using a different technique in each: the first using “Simulation and Modeling (vicarious experience)” of the disability; the second using “Information and Awareness Raising”; and the third using “Adapted Sport.”

## Materials and Methods

### Participants

The number of participants was determined, initially, from the 43,389 students registered in obligatory secondary education (ESO) in the Autonomous Community of Extremadura (Spain) in the 2018–2019 academic year, considering a sample error of 4% and a confidence interval of 96%. The total sample was made up of 548 secondary school students from 20 states and privately owned direct grant schools. These centers were selected at random by means of cluster sampling from a total of 120 centers. The study was carried out using one class group per school. Each school was assigned a particular year group (first, second, third, or fourth) so that all years would be represented. Assignation to experimental and control groups was carried out through a draw. As for the representativeness, 30% of the students were in their first year of secondary school, 27% in the second year, 22% in the third, and 21% in the fourth. Around 51.7% were girls and 48.3% were boys, all between 12 and 16 years of age *(M = 13.66; SD = 1.374)*.

### Instrument

#### “Brief Questionnaire for Adolescents on Attitudes Toward Persons With a Disability CBAD-12A”

This instrument (Álvarez et al., 2020) is chosen as it is the only instrument validated for evaluating attitudes toward persons from 12 to 16 years of age with a disability in the Spanish population. It is an instrument of great diagnostic and/or predictive utility when analyzing the attitudes of this age group. It is a brief instrument, created specifically for the need to have an evaluation instrument that can solidly analyze attitudes toward the said collective with sufficient evidence of validity and reliability, while also allowing a simple and fast application (Álvarez et al., 2020). The response format was of a Likert type with five possible answers, in which 1 is “totally disagree” and 5 “totally agree.” Higher scores in the questionnaire indicate more negative attitudes toward persons with a disability (Álvarez et al., 2020).

According to the authors, the analyses carried out confirm that the variables associated with the attitudes of adolescents toward persons with a disability can be grouped into three factors (acceptance–rejection, competence–limitation, and equal opportunities) made up of four well-defined, solid and relevant items with weights over 0.50 and with an adequate internal consistency. For instance, one item from each factor of the questionnaire is presented: Factor 1 acceptance/rejection: *“I would not go to a place where they know me accompanied by a person with a disability”*; Factor 2 competence/limitation: “*Persons with a disability, in many aspects, are like children”*; and Factor 3. equal opportunities: *“An unemployed person without a disability should be contracted before an unemployed person with a disability.”*

The instrument is subjected to an AFC and the correlations between the three factors indicate that they are clearly related. This, in turn, indicates that as the negative attitudes toward disability in one of the factors increase, they also increase in the other two. A structural equation analysis is carried out and the random resampling method is applied which allows us to verify that the latent variables in the three factors are well defined and adequately evaluated, strengthening the good psychometric characteristics of the CBAD-12A. In addition, a multi-group analysis is applied that confirms the equivalence of the factorial structure of the questionnaire with respect to gender. Finally, in order to contrast its nomological validity, and starting from the hypothesis that those subjects who maintain contact with persons with a disability will have more positive attitudes toward them, comparisons between subjects with and without contact were carried out to confirm the said hypothesis.

In this study, the reliability indices for the different factors were: acceptance/rejection, Cronbach’s Alpha (*α* = 0.82), and McDonald’s Omega (Ω = 0.84); competence/limitation (*α* = 0.77, Ω = 0.77); and equal opportunities (*α* = 0.74, Ω = 0.74).

To confirm whether the model found in the original validation study (Álvarez et al., 2020) adequately fit our data; we have used the goodness indices described in Table 1. As can be seen, the model is adequate and the fitness indices are optimum, showing evidence of reliability and validity for the generalization of our results.

**Table 1:** Goodness-of-fit indices for the proposed model, Questionnaire (CBAD-12A) by Álvarez et al. (2020).

Model	χ^2^	χ^2^*/df*	GFI	IFI	TLI	CFI	RMSR	RMSEA
3 factors	140.767	2.760	0.972	0.931	0.909	0.930	0.042	0.046

χ^2^, Chi-square statistic; χ^2^/df, Chi-square divided by the degrees of freedom; GFI, Goodness-of-fit index; AGFI, Adjusted goodness-of-fit index; IFI, Incremental fit index; TLI, Tucker–Lewis index; CFI, Comparative fit index; RMSR, Root of the mean square residual; and RMSEA, Root mean square error of approximation.

### Procedure

The study was carried out in the centers selected for the intervention, over 9 weeks of a school term during class time. The centers were asked to complete an informed consent form for the research, a document which expressly asks the educational center to accept its participation in the research.

The research took place in three phases, the first 2 weeks before the intervention to fill in the CBAD-12A questionnaire for the control group (CG) and the experimental groups (EG1, EG2, and EG3). Qualified personnel went to the centers at appointed times during tutorial hours to supervise the filling-in of the questionnaire, attending to any doubts that the students might have. The tutorial hour was chosen to facilitate the task being carried out in a relaxed atmosphere with the presence of the tutor. The presentation, completion, and collection of the questionnaire took approximately 40 min. In the second phase, the three intervention programs were applied and the participants were distributed at random among the three versions, EG1, EG2, and EG3. The students from the CG received their normal tutorial class. Each program had a team of three monitors with a disability to coordinate the activities. The third and last phase took place 2 weeks after the intervention itself, to complete the CBAD-12A questionnaire once more in the same tutorial hour and under the same conditions as in the first phase.

The intervention programs and their methodologies are singular: the first (EG1) uses the simulation and modeling technique (vicarious experience); the second (EG2) uses the information and awareness raising technique concerning disability; while the third (EG3) uses the adapted sport technique. Each program consists of three sessions carried out over 6 weeks, one session each 2 weeks. The work methodology is mainly based on group dynamics, videos and bibliographical material, role-playing, simulation, and cooperative learning.

The interventions were carried out in different areas of each center. The purpose of the simulation and modeling technique (EG1) was to encourage a change of attitudes by putting the students in place of persons with a disability, simulating different situations through the use of technical aids and the analysis of the problems of universal accessibility. The information and awareness raising technique (EG2) aimed to provide the students with information concerning the conceptual, legal, and practical contents referring to disability, in the hope of raising awareness of this collective’s rights. The objective of the adapted sport technique (EG3) was to provide the students with knowledge of adapted sports through practicing them, thus putting themselves in the position of persons with a disability. Table 2 shows details of the interventions carried out with these different techniques. There were no interventions with monitors for the control group (CG), nor was there direct or indirect contact with the students who made up this said group.

**Table 2:** Details of the interventions carried out with these different techniques.

Program 1. Simulation and Modeling Technique	Program 2. Information and Awareness Raising Technique	Program 3. Adapted Sport Technique
**First session**. Presentation of the program and the monitors. Technical aids (wheelchairs, crutches, walking sticks, and walking frames) to overcome the environmental barriers. Explanation of their use with PowerPoint, videos and real demonstrations by the monitors (modeling). Learning to use the technical aids in three teams with a monitor to coordinate the activity. Discussion concerning the experience. Monitors talk of their experience in relation to the use of the said technical aids in their life. **Second session**. Simulation of the disability. Itineraries around the center using the technical aids. Detection and analysis of the different barriers in the environment. Observation of the classmates who simulate the disability, empathizing with the difficulties of the collective. The monitors guide and advise the students during the simulations. Pooling the teams’ ideas concerning the experience, analyzing the barriers in the environment. The monitors talk of their own experience when faced with such difficulties in their lives, not so much because of their disability but from the point of view of a lack of accessibility. **Third session**. Role playing daily situations (shopping, using public transport or going to a café). Simulation of some of the limitations and difficulties that persons with a disability have in their social environment. Subsequent analysis and debate concerning the experience. The monitors explain their own experience in the said situations, telling real anecdotes to demonstrate the difficulties that social attitudes generate in their day to day lives.	**First session**. Presentation of the program and the monitors Disability, attitudes, and effects on persons with a disability. Debate format (concept of disability, the knowledge students have of persons with a disability, society’s and students’ attitudes, analyzing beliefs, stereotypes and prejudices and their influence on persons with a disability). The monitors share their own experience with the students concerning the difficulties they have found with respect to the social attitudes they have come across in their lives. **Second session**. Convention on the Rights of Persons with a Disability (Organización de las Naciones Unidas, 2006). Each team is led by a monitor who coordinates the activity. Each team selects and analyzes three fundamental rights from the CRPD. Guided discussion on the degree of fulfilment of the said rights and their importance for persons with a disability. The teams create an awareness raising campaign with a poster concerning the three rights worked on. **Third session**. Each team presents their awareness raising campaign. Together with the monitor from each team, the students agree on placing the posters in a visible location within the center in order to raise awareness among the educational community.	**First session**. Presentation of the program and the monitors. Adapted sport in its different modalities. BOCCA, Presentation and explanation of the sport, its adaptations for persons with a disability and the techniques used to play it. Three teams are formed, a monitor acting as the captain of each team. A tournament is organized and played. Finally, a guided discussion is held concerning the experience. **Second session**. Presentation and explanation of wheelchair basketball, its adaptations for persons with a disability and the technique used. Wheelchair practice is performed and three teams, each one captained by a monitor, are formed. The three teams participate in a tournament. Finally, a guided discussion is carried out concerning the experience. **Third session**. Presentation and explanation of wheelchair slalom, its adaptations for persons with a disability and the technique used. Through the different sessions, the students analyze the benefits of sport for persons with a disability, simulating access to physical sporting activity, putting the students in the place of the persons with a disability. They work on the knowledge and simulated practice of adapted sports, demonstrating and practicing alternatives in sport. Finally, a guided discussion is carried out concerning the experience.

### Design

A quasi-experimental methodology was used with a non-equivalent control group and pre- and post-measures in both experimental and control groups. This is precisely one of the most commonly used methodologies in educational research. In most empirical studies carried out in the educational sphere, the quasi-experimental methods have taken on a great importance due to the organizational and ethical difficulties of providing a rigorous control of the experimental method in the educational context and to the desire to achieve the explanatory effectiveness of the cause-effect relations which are characteristic of the said method. A quasi-experimental design consists in applying experimental designs to real situations (educational, family, social etc.), but it has its limitations. The two fundamental strategies for controlling whether a confounding variable may have affected the results of the study are to include a control group and to take measures both prior to and following the application of the intervention.

We worked with independent groups, one control group (CG) and three intervention groups (EG1, EG2, and EG3). EG1 received the intervention with the *“Simulation and Modeling”* program and was made up of 219 students. EG2 received the *“Information and Awareness Raising*” program and was made up of 164 students. EG3 received the *“Adapted Sport”* program and was made up of 60 students. The control group was made up of 105 students.

The dependent variable, attitudes toward disability, was measured following the application of the different programs. The control group did not receive the intervention and all the groups (experimental and control) completed the pre-test and post-test phases with the brief questionnaire on attitudes toward disability (CBAD-12A) by Álvarez et al. (2020).

### Data Analysis

We initially carried out reliability analyses (Cronbach’s Alpha and McDonald’s Omega), as well as a confirmatory analysis (CFA) of the instrument used. We then submitted the data to the Kolmogorov–Smirnov and Levene tests in order to determine the assumptions of normality and homoscedasticity. *p* > 0.05 was found in all the contrasts, thus justifying the use of the tests: ANOVA for independent samples, the Student *t*-test for related samples and covariance analyses (ANCOVA). We also used tests for the size of the effect (*η*^2^ and Cohen’s *d*). The statistical techniques were carried out using the statistical package SPSS, version 21 (IBM Corp, New York, NY, United States, 2012) and JASP Free, while the AMOS-21 program was used for the CFA.

## Results

First of all, in order to know whether the pre-test scores of the CBAD-12A enable an appropriate base for inter-group comparisons (control-experimental), thus reducing the possibility that the estimates associated to the independent variable may be due to other factors which have not been taken into account and to contrast the effectiveness of the intervention carried out, we performed inter-group pre-test (control/experimental) and intra-group (pre-test/post-test) comparisons (Table 3). Similarly, to complete the information provided through the application of the significance tests (ANOVA and Student *t*-test), the size of the effect was calculated using the η^2^ and Cohen’s *d* statistics.

**Table 3:** Inter-group analysis.

		CG		EG1		EG2		EG3		*F*	*p*-value	*η* ^2^
		*M*	*SD*	*M*	*SD*	*M*	*SD*	*M*	*SD*			
Pre-test	F1: Acceptance/Rejection	6.92	2.77	7.35	2.69	7.44	2.66	7.56	2.90	1.014	0.386	0.007
	F2: Competence	10.42	3.06	10.49	2.84	11.35	2.87	11.26	3.55	3.891	0.009	0.019
	F3: Opportunity	6.50	2.12	6.90	2.41	6.85	2.19	7.47	2.99	2.046	0.106	0.013
Post-test	F1: Acceptance/Rejection	7.10	3.20	6.66	2.33	6.60	2.65	6.88	2.71	0.984	0.400	0.005
	F2: Competence	10.55	3.51	8.70	2.60	9.54	3.26	9.11	3.40	10.16	0.000	0.047
	F3: Opportunity	7.29	2.46	6.24	2.04	6.72	2.63	6.66	2.52	5.548	0.001	0.026

One-factor ANOVA. CG, Control Group; EG1, Simulation; EG2, Awareness Raising; and EG3, Adapted Sport.

In the inter-group pre-test comparisons (control/EG1/EG2/EG3), the lack of any significant differences in the factors 1 “acceptance/rejection” and 3 “opportunities” (*p* ≤ 0.05) in the scores of the CBAD-12A demonstrates the equivalence between the groups as a good basis for comparison (Table 3). Significant differences (*p* = 0.009) are only found in the factor “competence.” The pair comparisons of Bonferroni show that the differences are established between the experimental groups EG1 and EG2 (*p* = 0.021).

On the other hand, in the inter-group post-test comparisons (control/EG1/EG2/EG3), the experimental groups obtain significantly lower scores (*p* < 0.001) in the factors 2 (competence) and 3 (opportunities) del CBAD-12A with medium effect sizes. As for factor 2 “competence,” the pair comparisons of Bonferroni show that the differences are established between the control group and EG1 (*p* < 0.001), the control group and EG2 (*p* = 0.050), and the control group and EG3 (*p* = 0.020), as well as between the experimental groups EG1 “Simulation” and EG2 “Awareness Raising” (*p* = 0.027). These differences had already been established in the pre-test. As for factor 3 “opportunities,” the pair comparisons of Bonferroni show that differences are only established between the control group and EG1 (*p* < 0.001).

**Figure 1:**
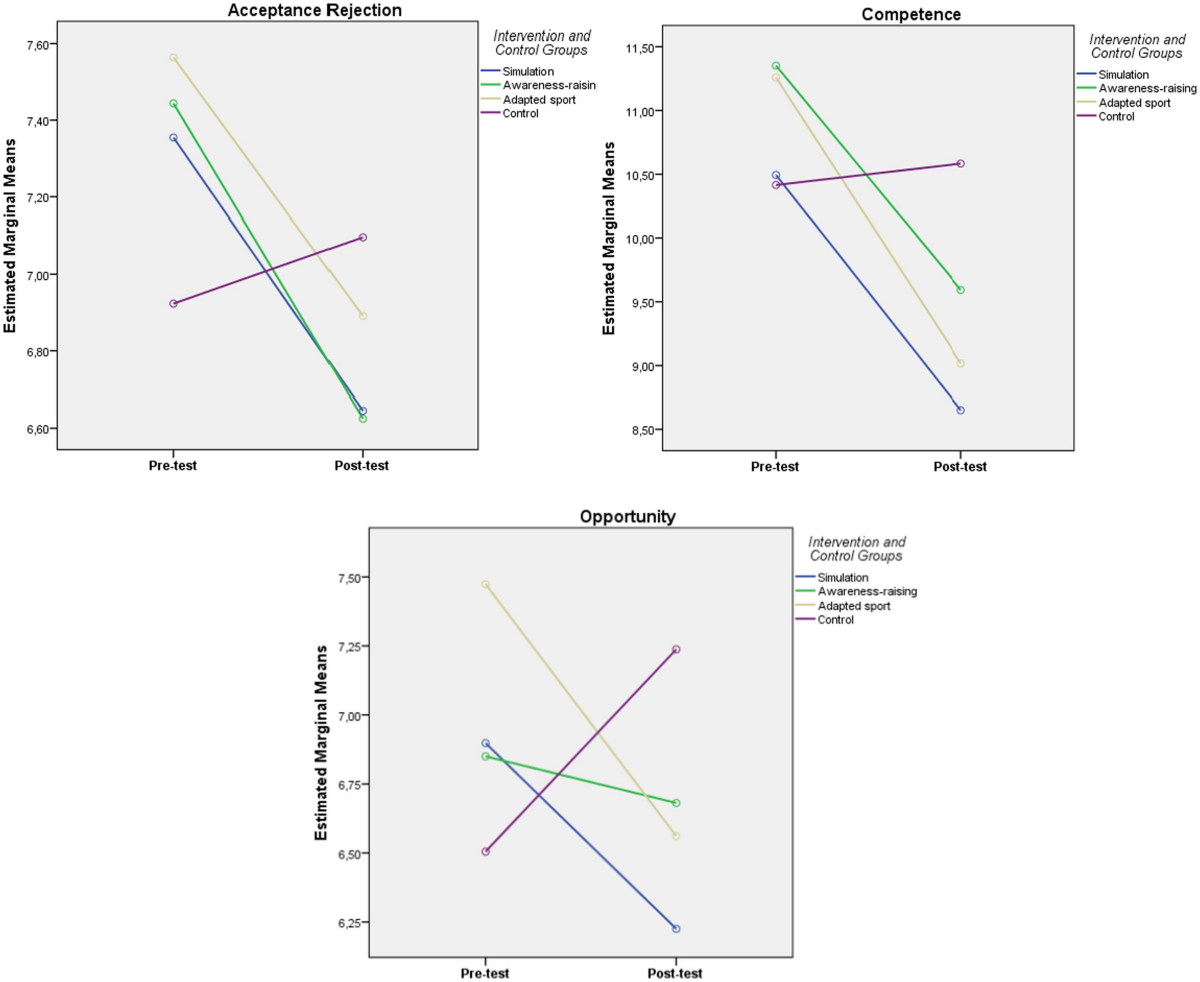
Results graphically Student *t*-test for related samples.

In order to control the possible variations due to differences between the control group and the experimental group, the data were subjected to the Student *t*-test for related samples (Table 4) so as to check whether the results found could be attributed to the independent variable (the intervention). Figure 1 shows these results graphically.

**Table 4:** Intra-group analysis.

		Pre-test		Post-test		*t*	*p*-value	*d*
		*M*	*SD*	*M*	*SD*			
CG	F1: Acceptance/Rejection	6.92	2.77	7.09	3.21	−0.525	0.601	0.057
	F2: Competence	10.41	3.06	10.58	3.57	−0.565	0.573	0.051
	F3: Opportunity	6.50	2.12	7.23	2.43	−2.845	0.005	0.332
EG1	F1: Acceptance/Rejection	7.35	2.69	6.64	2.32	5.571	0.000	0.283
	F2: Competence	10.49	2.84	8.65	2.50	12.596	0.000	0.689
	F3: Opportunity	6.90	2.41	6.22	2.03	4.411	0.000	0.306
EG2	F1: Acceptance/Rejection	7.44	2.66	6.62	2.67	3.481	0.001	0.309
	F2: Competence	11.35	2.87	9.59	3.25	8.301	0.000	0.576
	F3: Opportunity	6.85	2.19	6.68	2.61	0.854	0.394	0.071
EG3	F1: Acceptance/Rejection	7.56	2.90	6.89	2.72	1.906	0.062	0.240
	F2: Competence	11.26	3.55	9.02	3.37	5.132	0.000	0.653
	F3: Opportunity	7.47	2.99	6.56	2.49	2.100	0.040	0.334

Repeated measures *t*-Student.

For EG1 “Simulation,” the intra-group comparisons show a significant reduction (*p* < 0.001) between the pre-test and post-test scores in the three factors of the CBAD-12A, with medium to high size effects (*d* ≥ 0.69) in factor 2 “competence.” In EG2 “Awareness Raising,” significant differences (*p* ≤ 0.001) are found in factor 1 “Social Rejection” and factor 2 “competence,” with medium to high size effects (*d* ≥ 0.58) in factor 2 “competence.” For EG3 “Adapted Sport,” significant differences were found in factor 2 “competence” (*p* < 0.001) and factor 3 “opportunities” (*p* = 0.040), with medium to high size effects (*d* ≥ 0.65) in factor 2 “competence.” On the other hand, there is a significant increase (*p* = 0.005) within the control group between the pre-test and post-test scores in the factor 3 (opportunities), with a medium effect. This increase is contrary to our hypotheses.

The analysis of the size effects (Cohen’s *d*) provides evidence that the greater size occurs in factor 2 “competence” and also that, for factor 1 “Social Rejection,” the greater size effect occurs in EG2 “Awareness Raising” (*d* = 0.31). In factor 2 “competence,” the greater size effect occurs “Simulation” EG1 (*d* ≥ 0.69); while, for factor 3 “opportunities,” the greater size effect occurs in EG3 “Adapted Sport” (*d* = 0.33).

Finally, in order to control the effect of the regression to the mean, we have to eliminate the effect attributable to other variables not included in the design and thus not subject to control from the dependent variables (post-test scores of the CBAD-12) by carrying out inter-subject effect tests (ANCOVA). The pre-test scores of the dependent variables are used as co-variables and the different groups (control/EG1/EG2/EG3) as fixed factors.

The ANCOVA (Table 5) shows significant differences (*p* ≤ 0.05) between the groups (experimental and control) in factors 1 (acceptance/rejection), 2 (competence), and 3 (opportunities), confirming the results found in the inter-group comparisons (Table 2). So, the decreases observed in the scores of the CBAD-12A in the experimental groups may be attributable to the intervention carried out.

**Table 5:** Inter-subject effects test (ANCOVA).

		Origin	Sum of type III squares	*gl*	Quadratic mean	*F*	*p*-value	*η* ^2^
Post-test	F1: Acceptance/Rejection	Pre-test F1	1042.771	1	1042.771	202.727	0.000	0.254
		CG/EG1/EG2/EG3	39.698	3	13.233	2.573	0.049	0.013
		Error	3065.651	596	5.144			
	F2: Competence	Pre-test F2	1852.909	1	1852.909	313.981	0.000	0.345
		CG/EG1/EG2/EG3	310.089	3	103.363	17.515	0.000	0.081
		Error	3511.297	595	5.901			
	F3: Opportunity	Pre-test F3	434.497	1	434.497	94.183	0.000	0.136
		CG/EG1/EG2/EG3	104.524	3	34.841	7.552	0.000	0.037
		Error	2754.156	597	4.613			

CBAD-12A Factors/Group intervention.

## Discussion

This research aimed to analyze three intervention programs and their effectiveness in changing the attitudes toward persons with a disability of adolescent students in educational centers. Each of the programs had a particular technique as its base: “Simulation and Modeling” in program 1, “Information and Awareness Raising” in program 2 and “Adapted Sport” in program 3; all brought together by the technique of direct, structured contact with persons with a disability (Laws and Kelly, 2005; García and Hernández, 2011; Polo et al., 2011; Armstrong et al., 2017; Logan and Bogart, 2020; Kirk et al., 2021).

Concerning the first objective of this study, to analyze the effect of each program on the change in attitude toward persons with a disability, the results show the effectiveness of the interventions as the students demonstrate more positive attitudes toward this collective in all the groups (EG1, EG2, and EG3). This is not the case with the control group (CG), which maintains or even worsens their attitudes toward the collective in factor 3, “equal opportunities.” This may be due to the fact that, as they receive no interventions, they focus their perceptions on aspects related to the limitations (social prejudices and stereotypes) of the persons with a disability and not on their possibilities, capacities and potential (Bausela, 2009). On the basis of the consistency theory, it is considered that people make an effort to maintain a certain coherence in their beliefs, attitudes, and behavior (Briñol et al., 2003).

As for the effectiveness of the intervention programs, it was program “Simulation and Modeling” that, taking the three factors evaluated as a whole, achieved the greatest change in attitudes. Different studies consider the appropriateness of this technique. They pose the idea that, of the numerous techniques used to change attitudes toward persons with a disability, “simulation” is one of the most effective in promoting changes in attitudes (Slininger et al., 2000; Hodge et al., 2002; McCarthy and Light, 2005). Exposing students to the “simulation” of situations that persons with a disability have to face every day is one way for them to know such problems, directly, profoundly and “in person.” This will generate a greater empathy and a greater awareness of the competences required by persons with a disability when facing such tasks (Verdugo et al., 1994; López and López, 1997; Lean et al., 2006; Florez et al., 2009; Delgado, 2015; Seger et al., 2017). According to Ríos (2003), such an exposition encourages them to understand and evaluate the possible difficulties that persons with a disability have. It would seem much more effective to have a session in which the students sit in wheelchairs and visit their center, thus realizing that architectural barriers exist than to have a session presenting a series of slides concerning such architectural barriers. In short, the “Simulation and Modeling” program has allowed these students to live through certain situations that limit their normal functionality; an artificial situation that helps to educate the students in values and attitudes toward persons with a disability.

Nevertheless, some studies question this technique, as they consider it generates stress, pressure and a lack of confidentiality in the participants, while not promoting an improvement in attitudes toward persons with a disability (French, 1992; Kiger, 1992; Herbert, 2000; Silverman et al., 2015; Nario-Redmond et al., 2017). As for the research bases in this kind of questions concerning the technique of simulation, we are fully aware of the fact that, for a technique to be effective, the risks must be minimized and the planning of the activities has to be rigorous. Thus, in the simulation program carried out, the activities were always in groups supervised by monitors with a disability. The simulations were based on certain guidelines to guarantee their success, such as providing prior information to resolve any doubts; explaining the capacities of the monitors with a disability so as to resolve any problems they might face and thus prevent feelings on the part of the participants of despondency or considering the task to be impossible; informing of the possible risks and reminding the participants of the precautions they should take; anticipating the possibility of feelings of unease, allowing them to refuse to do the activity or to interrupt it at any moment, with the possibility of choosing the type of simulation in which they wish to participate; carrying out the activities in groups to face the limitations posed and to find solutions to the problems that arise; planning the experience with a sufficient duration of time in order to be able to understand and overcome the challenges that a disability sometimes presents; creating an atmosphere of seriousness; giving the participants time to interiorize the simulation; proposing how the lives of persons with a disability would change if they had the necessary support (accessibility), instead of focusing on the negative aspects and difficulties; combining the active development of the simulation with the observational participation of the companions; finishing the activity with a colloquium-debate and discussion directed by the monitors with a disability concerning their lived experiences, in order to analyze the situations they lived through and observed in others, thus enabling the sharing of information concerning the difficulties found and generating solutions to improve accessibility in the environment (Donaldson, 1987; Clunies-ross and O’meara, 1989; Pfeiffer, 1989; Behler, 1993; Verdugo et al., 1994; López and López, 1997; Goddard and Jordan, 1998; Burgstahler and Doe, 2004; Hurst et al., 2012).

The second aim was to analyze the impact of each one of the programs in the different factors of the attitude construct: factor 1 “acceptance/rejection,” factor 2 “competence/limitation,” and factor 3 “equality of opportunities.” In this sense, and following (Verdugo et al., 1994), it is important for the interventions to take into account all three components of attitudes, the cognitive, affective, and behavioral. Analyzing the general influence of the three programs on the factors (F1, F2, and F3), it can be appreciated that attitudes have improved significantly in factors 2 and 3 for all three programs of the intervention. The students of the experimental groups (EG) show a more positive perception of persons with a disability with respect to their competence and equality of opportunities (Slininger et al., 2000; Morton and Campbell, 2008; Florez et al., 2009; Xafopoulos et al., 2009; Linsay and Edwards, 2012). As for factor 2, all three programs show positive changes, especially in program “simulation,” which is the most effective in bringing about changes in attitudes. The students who participated in this program are the ones with the greatest perception of the competence of persons with a disability. According to recent studies, the Simulation and Modeling technique brings with it a positive modification in attitudes (Stathi et al., 2014; Ginevra et al., 2021). As for factor 3, “equality of opportunities,” all the groups improve their attitudes, especially those in program “Adapted Sport,” being the most effective in changing attitudes in the said factor. After participating in the program “Adapted Sport,” these students showed a greater awareness of equality of opportunities for persons with a disability (Kasser and Little, 2005; Reina et al., 2011).

The third objective was to evaluate how each of the intervention programs exerted influence with respect to the attitude factors. The results show that each of the programs achieved greater effectiveness in a particular factor. The “Simulation and Modeling” program generated a greater effect in factor 2 “competence/limitation.” Simulating a situation of disability and putting oneself in the place of a person with a disability, who has to face day to day life situations and their ensuing difficulties, has an important effect on one’s perception of the collective’s competence (Horne, 1988; Verdugo et al., 1994; Goddard and Jordan, 1998; Reina et al., 2010). According to Seger et al. (2017), exposing the students to the “simulation” of situations that persons with a disability normally have to face generates greater empathy and greater awareness of the competences required by persons with a disability to deal with such tasks.

The intervention program *“Information and Awareness Raising”* had real effects on F1 and F2, but not F3; it was this program which had a greater impact on factor 1 “acceptance/rejection,” the affective component of the attitude. These results coincide with other works of research in which the condition of empathy and emotional attachment is, without doubt, an important aspect that can be observed in the intervention programs (Asensio, 2002; Avramidis and Norwich, 2002; Moore and Nettelbeck, 2013). Providing knowledge concerning persons with a disability, information concerning the prejudices and stereotypes this collective suffers from and raising awareness of their rights have all had a positive impact on rapprochement to the collective. In this sense, the students show greater acceptance and adopt a more competent viewpoint toward persons with a disability. According to several works of research, these attitude changing programs can encourage the development of a greater social conscience in adolescents, leading to a more intense social acceptance of persons with a disability that breaks with the existing prejudices and stereotypes toward them (Aguado et al., 2004; Linsay and Edwards, 2012; Delgado, 2015; Gurdián et al., 2020; Lawson and Beckett, 2021).

The program that uses the technique of “Adapted Sport” is the one that shows a greater size effect in factor 3, “equality of opportunities.” Our data are coherent with other studies as far as the effectiveness of this intervention technique is concerned, since they demonstrate that adapted sport greatly encourages equality of opportunities and the raising of social awareness toward this collective in secondary school students (Robles-Rodríguez et al., 2017; Árnadóttir et al., 2018; Felipe et al., 2020).

An important point that should be taken into account following the analysis of the results is that the three interventions carried out all had the common thread of the technique of structured contact which showcases the capacities of the monitors with a disability through positive interactions, thus facilitating the acknowledgement of their potential as persons and generating more accurate expectations, modifying attitudes toward them (Laws and Kelly, 2005; Rillotta and Nettelbeck, 2007; Wong, 2008; Díez et al., 2011; Lemmer and Wagner, 2015; Cocco et al., 2020; Boin et al., 2021; Sadziak et al., 2021).

The contact technique developed in this study (in the three intervention programs) followed the premises and conditions provided by the research, included in the introduction section of this paper, in order to optimize the effectiveness. The role of protagonist given to the monitors with a disability in the three intervention programs is considered to be a fundamental factor as far as the effectiveness and the results are concerned. All the activities were coordinated and supervised by the team of monitors with a disability in each program, fulfilling the ideal requirements as competent models.

In this sense, similar results to those of our study indicate that the combined effect of various techniques would seem to be more effective when it comes to generating positive changes in attitudes toward persons with a disability in all its components (Aguado et al., 2004; Krahé and Altwasser, 2006; Wong, 2008; Xafopoulos et al., 2009; Ison et al., 2010; Reina et al., 2011; Santana and Garoz, 2013).

## Limitations and Future Lines of Research

As limitations of our study, we must list those of the quasi-experimental designs, which are derived from having worked with natural groups, thus being unable to assign the participating students randomly to the experimental or control groups. There is, therefore, no total control over the experimental variables and thus the results should be interpreted with caution. On the other hand, another limitation is the monitoring of the changes, the measurement of the effect of the different intervention modalities over the long term, carrying out different measurements to evaluate the maintenance of the change in attitudes.

Finally, the common thread of the technique of direct contact with persons with a disability and the techniques of the intervention programs themselves (information, simulation, and adapted sport) make it more difficult to be able to clearly and unequivocally attribute the effectiveness of the attitude change to the concrete techniques of each program. This could be overcome in the future by designing interventions in which the technique of “contact” (persons with and without a disability) and their interaction with the techniques of “information,” “simulation,” and “adapted sport” could be controlled.

As future lines of research, we would consider it relevant to analyze in greater detail the effects generated in the attitude change with respect to such alternative variables as the monitor (with or without a disability, type of disability), the type of intervention and the time, among others. Furthermore, we would like to continue working on the analysis of different aspects to improve the programs. Finally, we believe it would be useful to carry out further studies to analyze the effects and the consolidation of the changes in the long term.

## Conclusion

The modification of attitudes toward persons with a disability is complex and requires studies to analyze the most effective techniques to bring such a change to fruition. Being aware of the difficulty of the task, we believe it is important to bring the reality of persons with a disability closer to educational centers, to ages at which the negative attitudes toward the said collective are still not consolidated. This is essential for promoting more positive visions of the collective and to construct a more inclusive society, developing intervention programs that can translate this reality in a practical and “vivid” way (González and Baños, 2012; Felipe and Garoz, 2014; Harrison et al., 2019).

An important value of our research is the analysis of the effect of the programs posed and the differential effectiveness of the various components of the attitude construct, providing further knowledge in this respect (Verdugo et al., 1994). The techniques of simulation, information, and adapted sport demonstrate their effectiveness for changing attitudes. The study has shown that not all the techniques produce the same effect in the attitude construct. Attitude is a complex construct made up of different factors and our work poses the need to go deeper into a more detailed analysis of which programs and techniques affect most effectively these said factors. This will help to modify attitudes toward the collective in an effective and integral way. Finally, we consider that programs which bring together different techniques, such as those used in this research, would obtain more effective results in modifying the attitude construct in all its factors.

Despite the limitations of the study, it still provides a valuable contribution to the sphere of Inclusion and attitude change toward persons with a disability. We put forward new advances concerning the effectiveness of the techniques used and we are optimistic that the results obtained will lead to a change in attitudes on a social level, starting in our particular case from the secondary school stage, which is a key moment in a person’s development and in the consolidation of his/her attitudes. As stated by Aguado et al. (2004), if the positive attitudes decrease over the years, the benefit of interventions such as the one presented in this study is twofold, as not only have attitudes not worsened, they also do not return to their initial states. We can conclude that the application of well-designed and adequately planned intervention programs will produce positive effects in modifying the attitudes of adolescent students, which may lead to overcoming the obstacles created by prejudice toward persons with a disability, as well as encouraging respect for differences and acceptance of the collective as part of the diversity and equality of opportunities.

## Data Availability Statement

The original contributions presented in the study are included in the article/supplementary material, further inquiries can be directed to the corresponding author.

## Ethics Statement

The studies involving human participants were reviewed and approved by Ethics Committee of the University of Extremadura. Written informed consent to participate in this study was provided by the participants’ legal guardian/next of kin.

## Author Contributions

JÁ-D, BL-d-B, M-IP-d-R, SM-L, and VL-R contributed to conception, design of the work, analysis and interpretation of the data, and drafting the work. All authors contributed to the article and approved the submitted version.

## Funding

This research has been funded by the support to Research Groups of the Junta de Extremadura (SEJO14; GR21033) and Ministry of Economy Science and Digital Policy of the Junta de Extrema-dura and the European Social Fund (ESF).

## Conflict of Interest

The authors declare that the research was conducted in the absence of any commercial or financial relationships that could be construed as a potential conflict of interest.

## Publisher’s Note

All claims expressed in this article are solely those of the authors and do not necessarily represent those of their affiliated organizations, or those of the publisher, the editors and the reviewers. Any product that may be evaluated in this article, or claim that may be made by its manufacturer, is not guaranteed or endorsed by the publisher.
